# Top-down synthesis of polyoxometalate-like sub-nanometer molybdenum-oxo clusters as high-performance electrocatalysts†

**DOI:** 10.1039/c9sc05469c

**Published:** 2019-12-05

**Authors:** Rongji Liu, Kecheng Cao, Adam H. Clark, Peilong Lu, Montaha Anjass, Johannes Biskupek, Ute Kaiser, Guangjin Zhang, Carsten Streb

**Affiliations:** Institute of Inorganic Chemistry I, Ulm University Albert-Einstein-Allee 11 89081 Ulm Germany carsten.streb@uni-ulm.de rongji.liu@uni-ulm.de; CAS Key Laboratory of Green Process and Engineering, Institute of Process Engineering, Chinese Academy of Sciences 100190 Beijing China zhanggj@ipe.ac.cn; Central Facility of Electron Microscopy for Materials Science, Ulm University Albert-Einstein-Allee 11 89081 Ulm Germany; Paul Scherrer Institut Forschungsstrasse 111 Villigen CH-5232 Switzerland; University of Chinese Academy of Sciences 100049 Beijing China; Helmholtz-Institute Ulm, Electrochemical Energy Storage Helmholtzstr. 11 89081 Ulm Germany

## Abstract

The top-down fabrication of catalytically active molecular metal oxide anions, or polyoxometalates, is virtually unexplored, although these materials offer unique possibilities, for catalysis, energy conversion and storage. Here, we report a novel top-down route, which enables the scalable synthesis and deposition of sub-nanometer molybdenum-oxo clusters on electrically conductive mesoporous carbon. The new approach uses a unique redox-cycling process to convert crystalline Mo^IV^O_2_ particles into sub-nanometer molecular molybdenum-oxo clusters with a nuclearity of ∼1–20. The resulting molybdenum-oxo cluster/carbon composite shows outstanding, stable electrocatalytic performance for the oxygen reduction reaction with catalyst characteristics comparable to those of commercial Pt/C. This new material design could give access to a new class of highly reactive polyoxometalate-like metal oxo clusters as high-performance, earth abundant (electro-)catalysts.

## Abstract 2

The top-down synthesis and deposition of polyoxometalate-like clusters on porous carbon is reported together with the high electrocatalytic oxygen reduction reactivity of the composite.

## Graphical abstract

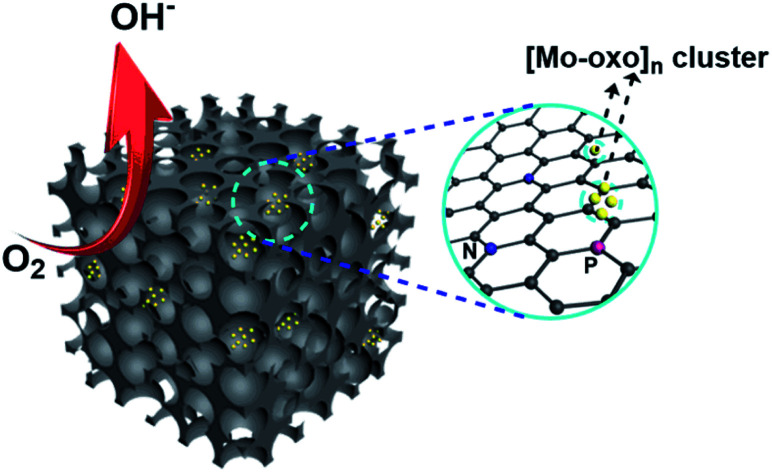

## Introduction

Many catalytic processes in energy conversion, such as electrolysis, fuel cells and metal–air batteries, rely on the stable immobilization of highly active metal reaction centers on electrically conductive high surface-area electrodes.^1–3^ A prime example is the oxygen reduction reaction (ORR) which is performed in commercial systems using precious metal catalysts such as platinum to accelerate the sluggish reaction kinetics. However, for large-scale deployment, *e.g.* in fuel cells, this results in significant costs due to the limited catalyst availability. In addition, platinum catalysts can undergo detrimental changes under operation and are “poisoned”, *e.g.* by CO or methanol.^4^ Therefore, earth-abundant transition-metal based catalysts, such as transition-metal oxides,^5,6^ carbides and nitrides,^7,8^ have been explored as technologically viable alternative ORR catalysts. However, the complexity of many of these catalytic composites together with a lack of suitable structural models to explore structure–function–activity–stability relationships is a significant challenge in the field.^9^

For electrocatalytic performance, the ideal scenario is the maximum dispersion of accessible reactive sites on the electrode surface. To this end, molecular metal oxides or polyoxometalates (POMs) are ideal candidates for this task as they are nanometer-sized reaction sites, which have been successfully used in electrochemical energy conversion and storage, including water electrolysis,^1,10–15^ batteries^1,16–18^ and metal–air batteries.^19,20^ POMs are ideally suited for these challenging applications, as their structure, composition and reactivity can be tuned on the molecular level,^21^ and they show remarkable performance even under chemically harsh conditions. In addition, POM-deposition on conductive substrates has been successfully established for the bottom-up design of electrocatalyst composites.^22,23^

Intriguingly, however, almost no POM-containing ORR catalysts have been reported to-date, despite the importance of metal oxides for the ORR:^24^ only recently, pioneering studies demonstrated the outstanding performance of polyoxotungstates as ORR catalysts in the context of metal–air batteries.^19,20^ In contrast, POMs have been used as molecular precursors for nanostructured metal oxides and carbides as highly efficient ORR electrocatalysts.^5,7,25,26^

This suggests that fundamental challenges related to the molecular nature of POMs prevent their wider development for ORR electrocatalysis: these challenges include poor mechanical and electrical linkage between POMs and electrodes, resulting in leakage during operation and high electrical resistance, *i.e.* poor catalytic performance.^22^ In addition, the wet-chemical bottom-up fabrication routes typically employed only allow deposition of solution-stable POMs and thus do not give access to more reactive species with higher ORR activity.^27^ Pioneering studies have started to address these challenges by developing new deposition routes: Laskin and colleagues have recently used a mass spectrometry-based ion soft landing technology to deposit mass-selected, electroactive POM anions from the gas phase directly onto electrode surfaces.^28,29^ In addition, Newton and colleagues have demonstrated that internal integration of POMs into conductive carbon nanotubes leads to remarkable improvement and stabilization of their electrochemical performance.^30^

Here, we propose an unprecedented top-down approach for the deposition of polyoxometalate-like metal oxo clusters on high-porosity carbon electrodes. The approach employs classical solution-stable Keggin polyoxomolybdates [H_3_PMo_12_O_40_] (= **PMo12**), which combine Brønsted-acidity, redox-activity and catalytic activity.^31^**PMo12** serves as a molecular precursor for the deposition of reactive sub-nanometer molybdenum(vi)-oxo clusters ([Mo-oxo]_*n*_, *n* = 1–20) on a high surface-area conductive mesoporous carbon electrode^32–34^ using redox-driven fragmentation and reassembly.^35–37^ A high Mo-oxo mass loading (>10 wt% Mo) together with a homogeneous molecular dispersion leads to outstanding electrocatalytic ORR performance comparable with that of noble metal references. Initial mechanistic studies highlight the characteristics of molecular metal-oxo species as well as their stability under catalytic turnover conditions. This work opens new, scalable material fabrication routes, which can give access to unprecedented molecular metal oxo cluster species for electrocatalysis.

## Experimental

### Synthesis of composite **1**

Composite **1** was synthesized using commercial silica gel powder with a pore size of 150 Å (Sigma-Aldrich) as a hard template. Typically, 2.555 g (1.4 mmol) of phosphomolybdic acid hydrate ([H_3_PMo_12_O_40_]·*x*H_2_O (=**PMo12**) Alfa Aesar) was dissolved in 5 g of water, and then 1.25 g (3.63 mmol) of sucrose (Merck Millipore (Calbiochem)) was added and dissolved. Finally, 1.0 g of silica powder was dispersed in the above solution and stirred overnight at room temperature. The mixture was heated in air to 100 °C for 6 h and subsequently to 160 °C for another 6 h. This impregnation process was repeated with a second solution containing 0.8 g (2.32 mmol) of sucrose and 1.643 g (0.9 mmol) of **PMo12** in 5 g of water. The air-dried materials were carbonized at 900 °C for 5 h under Ar at a heating rate of 5 °C min^−1^. To remove the silica template, the as-prepared composites were stirred in 50 mL of 10% aqueous hydrofluoric acid (HF_aq_, ≥ 48%, Sigma-Aldrich) for 48 h, and then washed with water and ethanol three times respectively, and finally dried at 100 °C overnight. During this step, **PMo12** was reduced and converted to MoO_2_, while the subsequent HF treatment did not significantly affect the chemical makeup of the molybdenum-based species in **1**. Note that hydrofluoric acid is toxic and necessary handling precautions need to be taken.

### Synthesis of composite **2**

0.1 g of **1** was dispersed in 10 mL of 10% aqueous HNO_3_ (∼65%, ACROS) and stirred for 5 hours at 50 °C. The powder was filtered off, washed with water (4×) and dried at 100 °C overnight, giving solid, dry composite **2**.

### Synthesis of composite **3**

Composite **2** (25 mg) was mixed with 0.25 g of 50 wt% aqueous cyanamide solution as a nitrogen source (99%, Aldrich) and 0.25 g water. The mixture was stirred overnight and left to dry at 30 °C in air for 48 h. The dried powder was firstly calcined at 550 °C for 4 h (heating rate 4 °C min^−1^) and then at 650 °C for 3 h (heating rate 4 °C min^−1^) in Ar to obtain composite **3**.

### Synthesis of reference samples

Mesoporous carbon (MC), oxidized mesoporous carbon (OMC) and nitrogen-doped mesoporous carbon (NMC) references were prepared following the same method as that of **1**, **2** and **3** respectively by using concentrated sulfuric acid (96%, ACROS) as the carbonization catalyst. Composite **4** was prepared by physical mixing (prolonged manual grinding) of NMC with commercial MoO_3_ (99.5%, Alfa Aesar, the loading amount of Mo was similar to the loading of **3**).

### Electrocatalytic ORR

2 mg of the finely ground catalyst (**1**, **2**, **3** or reference samples) was dispersed in 400 μL anhydrous ethanol ([catalyst] = 5 mg mL^−1^) by sonication for 1 h to form a homogeneous ink. Then 12.6 μL of the catalyst ink were loaded onto a glassy carbon (GC) rotating disk electrode (RDE) or a rotating ring-disk electrode (RRDE) with 4 mm diameter (the loading of the catalysts was 0.5 mg cm^−2^). To fabricate the working electrode of the Pt/C catalyst, 1 mg of catalyst (platinum on carbon black 20%, HiSPEC® 3000; Alfa Aesar) was dispersed in 1 mL of anhydrous ethanol (1 mg mL^−1^) using sonication for 30 min to form a homogeneous ink. Then 12.6 μL of the catalyst ink were loaded onto a glassy carbon RDE or RRDE (the loading of the catalyst was 0.1 mg cm^−2^). After drying, the electrodes were further modified with a thin film of Nafion by dropwise addition of 1.0 μL of 0.5 wt% Nafion solution (in isopropanol) onto the electrode surface. A standard three-electrode cell was used and was operated at room temperature. The prepared thin-film covered RDE or RRDE was used as the working electrode. Platinum foil was used as the counter electrode (CE) and an Ag/AgCl (3 M aqueous KCl) or Hg/HgO (1 M aqueous KOH) electrode was used as the reference electrode (Hg/HgO was used as the reference electrode for all the long-term chronoamperometry tests). The electrolyte, 0.1 M aqueous KOH, was saturated with ultrahigh-purity Ar for 30 min before the CV measurements. The oxygen reduction experiment was performed by saturating with ultrahigh-purity O_2_ for 30 min before the CV measurements. The steady-state polarization measurements for the ORR were obtained using RDE and RRDE techniques. The Ag/AgCl and Hg/HgO electrodes were referenced against the reversible hydrogen electrode (RHE) in all measurements. The referencing was performed based on the Nernst equation:*E*_RHE_ = *E*_Ag/AgCl_ + *E*^0^_Ag/AgCl_ + 0.059 pH;*E*_RHE_ = *E*_Hg/HgO_ + *E*^0^_Hg/HgO_ + 0.059 pH.For 0.1 M aqueous KOH, *E*_RHE_ = *E*_Ag/AgCl_ + 0.977 V and *E*_RHE_ = *E*_Hg/HgO_ + 0.883 V.

## Results and discussion

### Synthesis of composites **1**, **2** and **3**

**Scheme 1:**
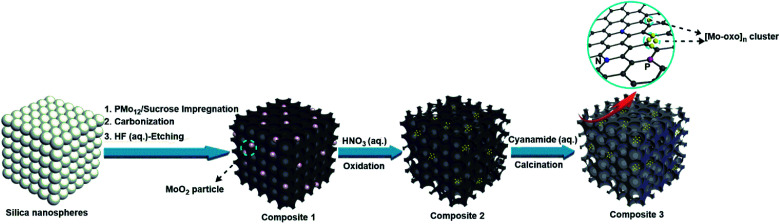
Fabrication route for composites **1**, **2** and **3**. Impregnation of a mesoporous silica hard template with the carbon source sucrose and the Brønsted acid and metal oxide precursor [H_3_PMo_12_O_40_] (**PMo12**) and subsequent carbonization and HF etching to remove the silica gives composite **1**, a mesoporous carbon containing Mo^IV^O_2_ nanoparticles. Subsequent oxidative fragmentation using aqueous HNO_3_ leads to the decoration of sub-nanometer [Mo-oxo]_*n*_ (*n* = 1–20) clusters on the carbon matrix of composite **2**. Subsequent cyanamide impregnation and calcination leads to the N-doping of the carbon matrix while retaining the [Mo-oxo]_*n*_ clusters in composite **3**.

The new composites are synthesized using a hard-templating route,^38^ where a commercial mesoporous silica template is impregnated with aqueous solutions containing sucrose as the carbon precursor and **PMo12** as the Brønsted-acidic carbonization catalyst and metal-oxo source (classical carbonization procedures employ sulfuric acid as the Brønsted acid; see the Experimental section for the synthesis of reference materials). Pyrolysis of the material was performed at 900 °C in an Ar atmosphere. The resulting black powder was washed with aqueous hydrofluoric acid (HF, 10 wt%) to remove the silica template, giving composite **1**. Subsequent oxidative treatment of **1** with diluted aqueous nitric acid (10 wt%) gave composite **2**. Impregnation of **2** with aqueous cyanamide solution (as a nitrogen source for N-doping of the carbon matrix) and subsequent calcination gave composite **3**, see Scheme 1.

### Characterization of composite **1**

**Fig. 1:**
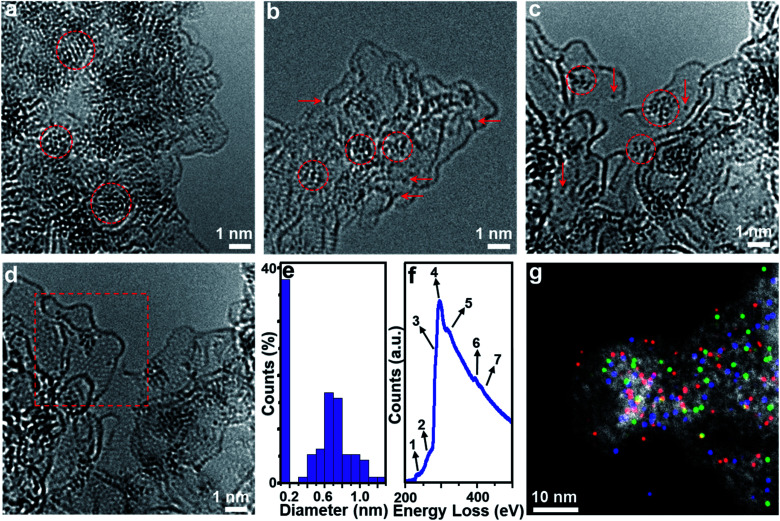
Morphologies and chemical structures of composites **1**, **2** and **3**. (a–d) Aberration-corrected HRTEM images of **1** (a), **2** (b) and **3** (c and d). In (b) and (c), the arrows and the circles show [Mo-oxo]_*n*_ clusters with different metal atoms. (d) shows the same area as that of (c) with different focal lengths to visualize the graphitic structure of the carbon in the framed area. (e) Size distribution histograms obtained from TEM images of **3**. (f) EELS spectra (background subtracted) recorded from the HAADF-STEM image. Peak assignments for elements: 1 = Mo-M_5_, 2 = Mo-M_4_, 3 = C-sp^3^, 4 = C-sp^2^, 5 = C-ELNES (carbon fine structure electron loss near the edge structure), 6 = Mo-M_3_, and 7 = Mo-M_2_/N–K. (g) HAADF-STEM and corresponding overlaid EDS mapping of **3**, showing the homogeneous dispersion of Mo (red), P (green) and N (blue).

**Fig. 2:**
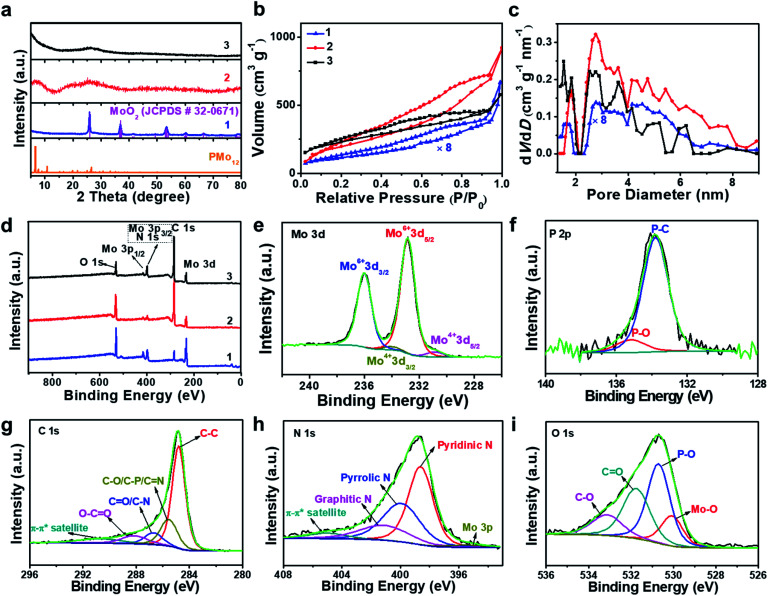
Structural characterization by PXRD, nitrogen sorption experiments and XPS. (a) PXRD spectra of **PMo12**, **1**, **2** and **3**. (b) Nitrogen sorption isotherms for **1**, **2**, and **3**. (c) The corresponding pore size distributions of **1**, **2** and **3**. (d) Survey XPS spectra of **1**, **2** and **3**. (e–i) High resolution deconvoluted XPS spectra for **3**, showing Mo 3d, P 2p, C 1s, N 1s and O 1s.

Aberration-corrected (AC) high-resolution transmission electron microscopy (HRTEM, Fig. 1a) indicates that **1** is based on layered carbon into which crystalline metal oxide particles (∼1–2 nm) are embedded. In addition, **1** contains larger MoO_2_ aggregates > 100 nm, which were observed by low-magnification TEM (Fig. S1†). Energy dispersive X-ray spectroscopy (EDS) elemental mapping combined with high-angle annular dark-field scanning transmission electron microscopy (HAADF-STEM) shows the homogeneous distribution of the elements Mo, P and O within the carbon matrix (Fig. S2†), and EDS quantification gives a Mo : O atomic ratio of ∼1 : 2, which is in line with the presence of MoO_2_ particles. Powder X-ray diffraction analysis of **1** (PXRD, Fig. 2a and S3†) indicated the presence of only one crystalline phase which was identified as MoO_2_ (JCPDS # 32–0671), which is in line with the TEM analysis. This indicates that during pyrolysis, **PMo12** (containing Mo(vi)) is reduced and converted to MoO_2_ (containing Mo(iv)).^39,40^ X-ray photoelectron spectroscopy (XPS) was used to assess the composition of **1**. The Mo 3d region (Fig. S4a†) was deconvoluted into four peaks assigned to Mo(iv) (in MoO_2_) and Mo(vi) (in MoO_3_, possibly formed by surface oxidation of MoO_2_).^41,42^ The P 2p spectrum was deconvoluted into two peaks assigned to P–C and P–O bonds (Fig. S4b†),^43^ suggesting that P is integrated in the carbon framework. For C 1s (Fig. S4c†), four peaks assigned to C–C, C–O/C–P, C=O and O–C=O are observed.^43^ The O 1s spectrum (Fig. S4d†) was deconvoluted into four signals assigned to Mo–O, P–O, C=O and C–O.^44^ For detailed XPS data, see Table S1.† Nitrogen sorption analyses using the Brunauer–Emmett–Teller (BET) method gave a specific surface area of 52.2 m^2^ g^−1^ (Fig. 2b and c, and for detailed BET analysis see ESI, Table S2 and Fig. S5†).

### Characterization of composite **2**

HRTEM analyses show that oxidative conversion of **1** into composite **2** results in major morphological changes (Fig. 1b and S6†). The surface of **2** is homogeneously decorated with metal oxo clusters with different sizes, while no MoO_2_ nanoparticles were observed. EDS mapping analysis with HAADF-STEM also shows the homogeneous distribution of the elements Mo, P and O within the carbon matrix (Fig. S7†). The PXRD analyses only give broad peaks assigned to the (002) and (101) planes of graphitic carbon and no peaks associated with crystalline MoO_2_ are observed, which is in line with the TEM analysis. In addition, **2** shows significantly higher porosity than **1** (BET surface area: 740.7 m^2^ g^−1^, see Fig. 2b, c and Table S2†). Based on XPS analyses (Fig. S8a†), the metal sites observed are mainly based on Mo(vi) species with minimal contributions from Mo(iv), indicating the efficient oxidation of MoO_2_ by the oxidative treatment performed. The deconvoluted P 2p and C 1s spectra are similar to the data observed for **1** (Fig. S8b and c†), indicating no significant changes in the chemical structure of the support. The broad O 1s peak (Fig. S8d†) can be deconvoluted into four peaks assigned to P–O, C=O, C–O and Mo–O bonds, indicating that the metal species observed in HRTEM are molybdenum-oxo aggregates. For comparison, the TEM images of MC based materials prepared by the classical procedures show no crystalline features or metal oxo clusters (Fig. S9†).

### Characterization of composite **3**

HRTEM analyses show that the morphology of composite **3** is essentially identical to that of composite **2** (Fig. S10a†). Molybdenum-oxo ([Mo-oxo]_*n*_, *n* = 1–20, see ESI note 1† for the determination of *n*) clusters with different sizes are observed on the single- or few-layer carbon support (Fig. 1c). The graphitic nature of the carbon is shown in Fig. 1d (framed area).

Size distribution analyses of the [Mo-oxo]_*n*_ clusters in **3** (see the histogram in Fig. 1e) show diameters of 0.4–1.3 nm (average diameter: 0.8 nm) with Poisson distribution. Furthermore, aberration-corrected HAADF-STEM of **3** also shows the different sizes of the [Mo-oxo]_*n*_ clusters which were in line with the HRTEM analyses (Fig. S10b†). Electron energy-loss spectroscopy (EELS) confirms the metal centers observed as molybdenum (Fig. 1f and S10c†). Combined EDS/HAADF-STEM mappings show the homogeneous distribution of the elements Mo, P, N and O within the carbon matrix (Fig. 1g and S10d†). Nitrogen sorption isotherm studies of **3** show high porosity (BET surface area: 822.7 m^2^ g^−1^, Fig. 2b and c, and for details see Table S2†).

PXRD of **3** shows similar features to those observed for **2** (Fig. 2a), indicating the presence of graphitic carbon and the absence of crystalline molybdenum oxide phases. XPS analyses of **3** (Fig. 2d) show the presence of Mo, O, C, N and P. The deconvoluted Mo 3d region indicates the presence of Mo(vi) and small amounts of Mo(iv) (Fig. 2e). The deconvoluted P 2p spectrum (Fig. 2f) also shows P–C features, suggesting that P is incorporated into the carbon framework. This is in line with the findings for **1** and **2**, and supported by EDS mapping (Fig. 1g). The deconvoluted C 1s spectrum (Fig. 2g) is similar to the spectra observed for **1** and **2**.^43^ The deconvoluted N 1s spectrum (Fig. 2h) shows three main peaks assigned to pyridinic, pyrrolic and graphitic N, suggesting that N was also doped into the carbon framework in **3**,^45^ which is considered advantageous for enhancing the conductivity and electrocatalytic activity of the material.^46^ The deconvoluted O 1s spectrum is nearly identical to the spectrum found for **2**,^44^ indicating the integrity of the [Mo-oxo]_*n*_ clusters which were also observed by HRTEM (Fig. 1c). For XPS details, see Table S1.† Furthermore, inductively coupled plasma optical emission spectroscopy (ICP-OES) analyses were performed and gave Mo loadings of 68.0 wt% (**1**), 10.3 wt% (**2**) and 10.4 wt% (**3**), respectively. The relative Mo contents in all these samples agree well with the above characterization.

### X-ray absorption spectroscopic analysis of **1**, **2** and **3**

**Fig. 3:**
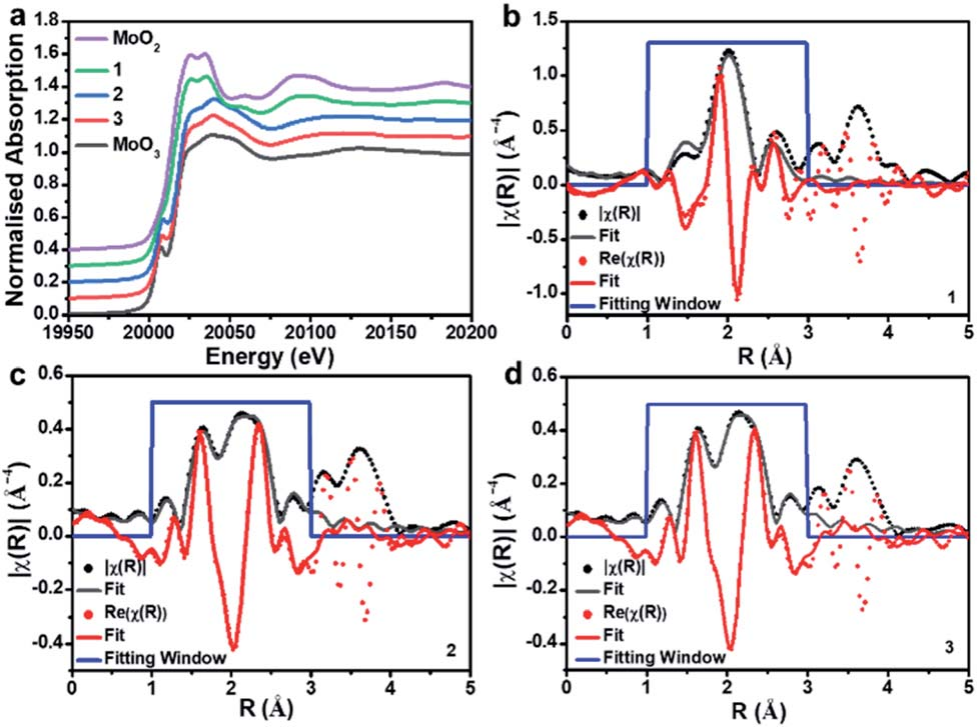
Mo K-edge XAS data. (a) Stacked plot of the XANES data comparing the three samples with MoO_2_ and MoO_3_ references. (b–d) Fitting to the R-space of the Mo K-edge EXAFS data for composites **1**, **2** and **3** showing the magnitude (black) and real components of R (red).

To gain further insights into the metal oxidation states and coordination environments in the three composites, we performed X-ray absorption spectroscopy (XAS) analyses at the SuperXAS beamline of the Swiss Light Source SLS. *Ex situ* XAS experiments were performed at the Mo K-edge of composites **1**, **2** and **3**. MoO_2_ and MoO_3_ were used as reference materials to probe the Mo oxidation state and the local coordination geometry. A stacked plot of the XANES (X-ray absorption near edge structure) data along with fitting to the R-space of EXAFS (Extended X-ray absorption fine structure) for the three samples is shown in Fig. 3. From XANES it is evident that **1** closely resembles the MoO_2_ reference and indicates Mo(iv) as dominant species. In contrast, **2** and **3** resemble the MoO_3_ reference, meaning that Mo(vi) is the dominant species; see Table S3† for XANES linear combination fitting.

EXAFS analysis (Table 1) shows that the first coordination shell for composite **1** is based on six-coordinated Mo–O with *d*(Mo–O) = 2.05 Å. This is in line with bulk MoO_2_.^47^ No interaction between the Mo and the carbon support is discernible in **1.** For **2** and **3**, a significant splitting in the first coordination shell is observed; Mo–O is found with two different distances, *ca.* 1.7 Å and *ca.* 1.9 Å. These distances are in agreement with those in POM-like Mo(vi) oxo clusters, which typically feature shorter terminal Mo=O bonds (∼1.7 Å) and longer, bridging Mo–O bonds (∼1.9 Å).^48^ Note that these results are in agreement with the XANES analysis above. For **2** and **3**, we observe a weak Mo–P interaction which could indicate bonding between Mo and the P-doped carbon support and may also be associated with some amorphous, P-rich aggregates observed in the EDS mapping (see Fig. S7a†). These questions will be explored further in future studies.

**Table 1:** Results of the EXAFS fitting to the Mo–K edge data

Composite	Scattering path	*N* a	*R* b (Å)	*σ* ^2^
**1**	Mo–O	6.0	2.05	0.010
	Mo–Mo	0.6	2.58	0.008
**2**	Mo–O	3.4	1.73	0.017
	Mo–O	1.4	1.93	0.012
	Mo–P	0.4	2.23	0.003
	Mo–Mo	0.4	2.67	0.008
**3**	Mo–O	4.1	1.73	0.017
	Mo–O	1.4	1.94	0.012
	Mo–P	0.4	2.22	0.003
	Mo–Mo	0.3	2.63	0.008

a Errors in the coordination numbers (*N*) are given as approximately 10%.

b Errors in the radial distance (*R*) are given as approximately 1%.

### ORR activity of composites **1**, **2** and **3**

**Fig. 4:**
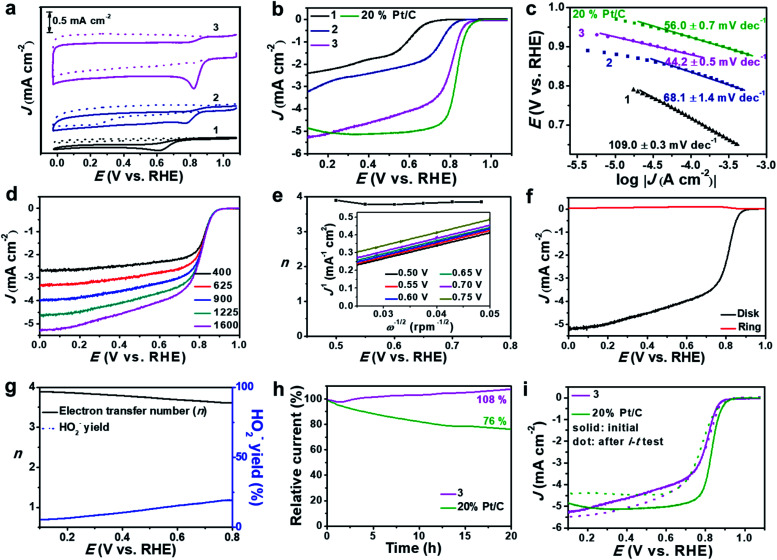
ORR activity studied by using RDE and RRDE techniques. (a) CV curves of **1**, **2** and **3** in Ar- (dotted line) or O_2_-saturated (solid line) 0.1 M aqueous KOH at a sweep rate of 5 mV s^−1^. (b) LSV curves of **1**, **2**, **3** and 20 wt% Pt/C in O_2_-saturated 0.1 M aqueous KOH at a sweep rate of 5 mV s^−1^ and a rotating speed of 1600 rpm. (c) The corresponding Tafel plots derived from the LSV data; note that the low overpotential region was used for Tafel analyses. (d) LSV polarization curves of a **3**-modified RDE in O_2_-saturated 0.1 M aqueous KOH at a sweep rate of 5 mV s^−1^ with varying rotation rates (400–1600 rpm). (e) Electron transfer number (*n*) for a **3**-modified RDE at different potentials obtained from *K*–*L* plots (see inset). (f) RRDE voltammograms recorded in O_2_-saturated 0.1 M KOH at 1600 rpm. The disk potential was scanned at 5 mV s^−1^ and the ring potential was constant at 1.45 V. (g) Yield of the peroxide species and the electron transfer numbers for **3** at different potentials (calculated from RRDE voltammograms). (h) Chronoamperometry of **3** and 20% Pt/C modified RDEs at 0.7 V *vs.* RHE in 0.1 M aqueous KOH with continuous O_2_ bubbling at 200 rpm. (i) LSV curves of **3** and 20% Pt/C before and after the *i*–*t* test.

Based on the high dispersion of the redox-active [Mo-oxo]_*n*_ clusters on high surface-area carbon, the electrocatalytic ORR activity of the composites was investigated and compared with that of reference samples (see the Experimental section for reference preparation). To this end, we deposited composites **1**, **2** or **3** on glassy carbon rotating disk electrodes (RDEs) and performed cyclic voltammetry in Ar- and O_2_-saturated alkaline electrolytes (0.1 M aqueous KOH, Fig. 4a and S11a†).

The studies show striking activity differences: for **3**, we observed the highest catalytic activity with the most positive oxygen reduction peak potential (*E*_p_ = 0.82 V) and the highest peak-current density (*J*_p_ = −0.94 mA cm^−2^). In contrast, both **1** and **2** showed significantly lower performance: for **1**, we observed *E*_p_ = 0.62 V and *J*_p_ = −0.30 mA cm^−2^, and for **2** we found *E*_p_ = 0.78 V and *J*_p_ = −0.31 mA cm^−2^ (Fig. 4a). For detailed comparison with all references, see Table S4.†

Notably, we observed superior performance also when comparing the activity of **3** with that of literature-reported ORR catalysts based on crystalline MoO_3_,^49,50^ highlighting that the high dispersion of [Mo-oxo]_*n*_ clusters on high surface-area carbon leads to improved ORR electrocatalysts. Moreover, composite **3** showed better or similar electrocatalytic ORR activities compared with reported metal oxide clusters or even noble metal (*e.g.* Ag, Au, Cu, *etc.*) clusters.^51–54^ Next, we used linear sweep voltammetric (LSV) measurements in O_2_-saturated 0.1 M aqueous KOH to compare the ORR activity of **3** with that of **1**, **2** and a commercial 20% Pt/C catalyst. As shown in Fig. 4b, composite **3** shows significantly more positive onset (*E*_onset_) potential as well as higher current density compared with **1** and **2** (Table 2). The comparison of **3** with a commercial 20 wt% Pt/C reference shows that the reference gives slightly superior ORR performance, see Fig. 4b and Table 2. The comparison of **3** with other reference samples is given in Fig. S11b.†

**Table 2:** Comparison of the electrocatalytic ORR activity of **1**, **2**, **3** and a 20% Pt/C reference

Samples	*E*_onset_ (V *vs.* RHE)	*J*_k_ (mA cm^−2^)	Tafel slope (mV dec^−1^)	ECSA (cm^2^)	*R*_ct_ (ohm)
**1**	0.75	10.17	109.0 ± 0.3	16 ± 1	108.8 ± 2.0
**2**	0.88	7.91	68.1 ± 1.4	44 ± 3	65.4 ± 1.8
**3**	0.93	20.02	44.2 ± 0.5	515 ± 25	53.1 ± 1.2
20% Pt/C	0.97	36.67	56.0 ± 0.7	277 ± 6	—

Based on the RDE-LSV data, we calculated the Tafel slopes (Fig. 4c) to gain insight into the ORR reaction kinetics: composite **3** shows significantly lower Tafel slopes than **1**, **2** and 20% Pt/C (Table 2), highlighting that the overall ORR-catalytic performance of **3** is comparable to that of the 20% Pt/C reference. To eliminate any leaching effects of platinum from the counter electrode to the working electrode during the ORR test,^55^ we also performed the catalytic studies of **3** by using a glassy carbon rod as counter electrode (Fig. S12†). No significant differences in the LSV curves are observed for both sets of experiments, showing that the presence of the Pt electrode does not affect the catalyst.

One major challenge in ORR electrocatalysis is the undesired 2-electron oxygen reduction, leading to the formation of peroxide species, which can cause damage to the electrode and catalyst.^56^ To assess the peroxide formation by **3**, we determined the electron transfer number (*n*) per oxygen molecule during the ORR based on RDE voltammetry (Fig. 4d). Based on Koutecky–Levich (*K*–*L*) plots, we calculated *n* at different potentials (Fig. 4e) and observed that **3** favors a clean 4-electron reduction of oxygen to water (*n* = 3.8–3.9 at *E* = 0.50–0.75 V). This was further verified by rotating ring disk voltammetry (RRDE, Fig. 4f) which supported a dominant 4-electron pathway (*n* = 3.6–3.9 at *E* = 0.10–0.80 V) and gave peroxide formation < 20% (Fig. 4g). Based on the RDE data, we calculated the kinetic current density (*J*_k_, based on the geometric surface area) at *E* = 0.5 V. For **3**, we found that *J*_k_ = 20.02 mA cm^−2^ while for 20% Pt/C, *J*_k_ = 36.67 mA cm^−2^. In contrast, composites **1** and **2** show significantly lower *J*_k_ and favor 2-electron pathways leading to peroxide formation (see Table 2, Fig. S13 and S14†). This further supports the high reactivity and selectivity of **3** compared to its precursors.

The long-term catalytic stability of **3** was evaluated by chronoamperometry (*i*–*t* test) at *E* = 0.70 V (Fig. 4h). In the initial phase (0–1.5 h), we note a minimal loss of activity (−2%); after this, the system shows increasing current densities, reaching values of 108% after 20 h operation. This can be described as an activation of the catalyst under operation. After this, the system shows no larger current density changes over a total period of 50 h (Fig. S15a†). In contrast, the 20% Pt/C reference sample shows a loss of activity of ∼24% over a period of 20 h. LSV analysis before and after *i*–*t* tests (Fig. 4i) shows negligible half-wave potential (*E*_1/2_) changes but a slight increase of current density for **3**, while for the 20% Pt/C reference a negative shift of *E*_1/2_ and reduced current density are observed due to the harsh cycling conditions. Further stability analyses were performed by an accelerated degradation test based on 3000 CV cycles (*E* = 0.60–1.0 V). LSV analysis before and after this test (Fig. S15b†) further confirms the high stability of **3**. Furthermore, **3** is highly tolerant to methanol, while most commercial Pt catalysts are poisoned by methanol in alkaline electrolytes (Fig. S16†), thus opening the door for further study of **3** as an ORR catalyst in direct methanol fuel cells.

To gain mechanistic insights into the superior ORR performance of **3**, we determined the electrochemically active surface area (ECSA) and the charge-transfer resistance (*R*_ct_) at the electrode/electrolyte interface, as these two parameters are critical for the reactivity of heterogeneous electrocatalysts. To this end, we used electrochemical double-layer capacitance (*C*_dl_, Fig. S17†) determination to obtain ECSA values and electrochemical impedance spectroscopy (EIS, Fig. S18†) to assess the *R*_ct_ of **1**, **2** and **3**. As summarized in Table 2, we observe significantly higher *C*_dl_ values for **3** compared with **1** and **2**, which is in line with the general specific surface area trends observed (see above). In addition, the subnanometer dispersion of the [Mo-oxo]_*n*_ clusters on electroactive N, P-doped carbon further improves the electrochemical performance. EIS supports this finding and shows that **3** features significantly lower charge-transfer resistances (Table 2), which enable efficient and fast electron transfer at the solid–electrolyte interface.

## Conclusions

In conclusion, we report a new design principle, which enables the simultaneous, homogeneous dispersion of polyoxometalate-like sub-nanometer molybdenum-oxo clusters on high-surface area carbon electrodes. The composites are prepared by a two-step hard-templating–redox cycling process where intermediately formed MoO_2_ nanoparticles on a carbon substrate are oxidatively dispersed to give sub-nanometer molybdenum-oxo clusters, which are stably linked to a high surface area N, P-doped graphitic carbon. The composite material performs as a stable and highly active oxygen reduction reaction catalyst and shows performance characteristics comparable with those of commercial Pt/C references. Based on our initial analyses, the high performance is correlated with the high dispersion of molybdenum-oxo clusters that act as stable, accessible and highly reactive surface sites. The high surface-area and porosity of the carbon substrate enable efficient mass transport and ensure unrestricted access to the catalytic sites. Finally, the N-doping of the carbon substrate enhances the electrical conductivity and promotes efficient charge transfer. Future studies will expand this blueprint to other reactive POM classes to enable deposition of earth-abundant (mixed) metal oxo clusters on carbon substrates. This could lead to a new class of high-performance electrocatalysts for challenging (proton-coupled) multi-electron redox-reactions.

## Conflicts of interest

There are no conflicts to declare.

## Supplementary Material

SC-011-C9SC05469C-s001
