# Supplementary material for: Top-down synthesis of polyoxometalate-like sub-nanometer molybdenum-oxo clusters as high-performance electrocatalysts
Source: Chem Sci. 2019 Dec 5;11(4):1043–51. doi: 10.1039/c9sc05469c (PMC8146420; doi:10.1039/c9sc05469c)
Supplement: SC-011-C9SC05469C-s001 [file SC-011-C9SC05469C-s001.pdf]

Electronic Supplementary Material (ESI) for Chemical Science.
This journal is © The Royal Society of Chemistry 2019

Electronic Supplementary Information

Top-down Synthesis of Polyoxometalate-like Sub-Nanometer Molybdenum-Oxo Clusters as High-Performance Electrocatalysts

Rongji Liu,*[ab] Kecheng Cao,[c] Adam H. Clark,[d] Peilong Lu,[be] Montaha Anjass,[af] Johannes Biskupek,[c] Ute Kaiser,[cf] Guangjin Zhang,*[b] and Carsten Streb*[af]

[a]Ulm University, Institute of Inorganic Chemistry I, Albert-Einstein-Allee 11, 89081 Ulm, Germany.

[b]CAS Key Laboratory of Green Process and Engineering, Institute of Process Engineering, Chinese Academy of Sciences, 100190, Beijing, China.

[c]Central Facility of Electron Microscopy for Materials Science, Ulm University, 89081 Ulm, Germany.

[d]Paul Scherrer

Institut, Forschungsstrasse 111, Villigen, CH-5232, Switzerland

[e]University of Chinese Academy of Sciences, 100049, Beijing, China.

[f]Helmholtz-Institute Ulm for Electrochemical Energy Conversion, Helmholtzstr. 11, 89081 Ulm, Germany.

*Emails: carsten.streb@uni-ulm.de; rongji.liu@uni-ulm.de; zhanggj@ipe.ac.cn

Table of Contents

1. Experimental Methods
2. Supplementary Note 1
3. Supplementary Data, Figures S1 to S13, Tables S1 to S5
4. Supplementary References

1. Experimental Methods

TEM and HRTEM measurements were performed using an image-side aberration corrected FEI Titan 80-300 at 80 kV accelerating voltage. Electron energy loss spectra were collected using a Gatan GIF 965 spectrometer.

HAADF-STEM and **EDS** were performed using a probe-corrected JEOL JEM-ARM200F TEM/STEM at 100 kV accelerating voltage and a Thermofisher Talos 200X equipped with a high dedicated high efficient EDS detector (0.9 sr collection angle) at 200 kV accelerating voltage. Both systems were also used to acquire local EDS mappings.

PXRD studies were performed on a BRUKER D8 Advance XRD unit using Cu−Kα (λ = 1.54 Å).

XPS analysis was performed on ESCALAB250 Thermo Electron Corporation equipment with an Al Ka X-ray source (1486.6 eV). The X-ray source was run at a reduced power of 150 W, and the pressure in the analysis chamber was maintained at $<10^{-11}$ Pa.

ICP-OES was performed on a Perkin Elmer Plasma 400 spectrometer. CV and LSV experiments were performed on a CHI 730E electrochemical workstation (CH Instruments Inc.).

EIS experiments were performed on a CHI 760E electrochemical system (CH Instruments Inc.) in the frequency range from 1000 kHz to 0.01 Hz with modulation amplitude of 5 mV.

XAS analysis: Ex-situ Mo K-edge XAS experiments were carried out at the SuperXAS beamline of the Swiss Light Source. The storage ring operated at 2.4 GeV in top-up mode with a ring current of 400 mA. The polychromatic X-ray beam resulting from a 2.9 Tesla bending magnet was collimated by a Pt-coated mirror at 2.5 mrad (which also served to reduce higher-order harmonics) and subsequently monochromatised by a Si(111) channel-cut monochromator. Data were collected in transmission geometry using the quick-scanning extended X-ray absorption fine-structure spectroscopy (QEXAFS) mode at 1 Hz monochromator oscillation frequency. Ionization chambers 15 cm long were filled with 2 bar N_2-filled. A Mo reference foil mounted between the second and third ionization chambers was measured simultaneously for absolute energy calibration. The samples were prepared as 13 mm pellets with the MoO_2, MoO_3 and sample **1** prepared with approximately 20 mg diluted with 40 mg of cellulose. Sample **2** and **3** were prepared with 100 mg of sample. The QEXAFS spectra were processed using a bespoke python program to extract individual XAS spectra, calibrate, normalise and average 5 minutes of data.

2. Supplementary Note 1

Electrocatalytic ORR

In alkaline solutions, the reduction of O_2 can proceed by two pathways:[1]

(1) Direct O_2 reduction to OH^- ions, the direct four-electron pathway (I):

$$O_2+2H_2O+4e^-\rightarrow 4OH^- \qquad (I)$$

(2) O_2 reduction to HO_2^- ions, a two-electron pathway (II):

$$O_2+H_2O+2e^-\rightarrow HO_2^-+OH^- \qquad (II)$$

followed by either an additional two-electron reduction of peroxide ions to OH^- ions (III):

$$HO_2^-+H_2O+2e^-\rightarrow 3OH^- \qquad (III)$$

or the decomposition reaction (IV):

$$2HO_2^-\rightarrow 2OH^-+O_2 \qquad (IV)$$

In order to establish the exact pathway of oxygen reduction involved at a particular electrode surface, RDE experiments were performed to examine the kinetics of ORR. For this purpose, RDE-LSV experiments were performed and the working electrode was scanned cathodically at a rate of 5 mV s^{-1} with varying rotating speed from 400 rpm to 1600 rpm. Koutecky–Levich (K-L) plots obtained from the LSV curves (J^{-1} vs. $\omega^{-0.5}$) were analyzed at various electrode potentials. The slopes of their linear fit were used to calculate the transferred electron number (n) per oxygen molecule involved in the oxygen reduction at each of the electrodes on the basis of the K-L equation:[2]

$$\frac{1}{J}=\frac{1}{J_k}+\frac{1}{B\omega^{0.5}}$$

where J is the measured current density at a given potential, J_k is the kinetic current density, and ω is the electrode rotating rate. Therefore, 1/B is the calculated slope and its unit is mA^{-1} cm^2 $rpm^{0.5}$. B could be determined from the slope of K-L plots based on the Levich equation as follows:

$$B=0.2nF(D_{O_2})^{2/3}\nu^{-1/6}C_{O_2}$$

where n represents the number of electrons transferred per oxygen molecule, F is the Faraday constant (F = 96485 C mol^{-1}), D_{O_2} is the diffusion coefficient of O_2 in 0.1 M KOH (1.9 ×10^{-5} cm^2 s^{-1}), ν is the kinetic viscosity (0.01 cm^2 s^{-1}), and C_{O_2} is the bulk concentration of O_2 (1.2×10^{-6} mol cm^{-3}). The constant 0.2 is adopted when the rotation speed is expressed in rpm.[3]

The RRDE technique is another efficient method to estimate the electron transfer number (n), in which the peroxide species produced at the disk electrode were detected by the ring electrode. For this purpose, the LSV was recorded and the disk electrode was scanned cathodically with a rotation speed of 1600 rpm at a scan rate of 5 mV s^{-1}, and the ring potential was constant at 1.45 V vs. RHE. The % HO_2^- and n were calculated from the ratio of the ring current (I_r) and the disk current (I_d) following the equations given below:[4]

$$\%HO_2^- = 200 \times \frac{I_r/N}{I_d + I_r/N}$$

$$n = 4 \times \frac{I_d}{I_d + I_r/N}$$

where N is current collection efficiency of the Pt ring (0.4).

Calculation of the maximum number of Mo centers

The standard MoO_3 (molybdite) crystal lattice has a unit cell volume V_{UC} = 0.228 nm^3,[5] each unit cell contains 4 Mo centers, so that per 1 nm^3, there are ~17.5 Mo centers. Thus, for the maximum observed particle size (1.3 nm diameter, $V_{particle}$ ~1.15 nm^3), there are approximately 20 Mo atoms.

3. Supplementary Data

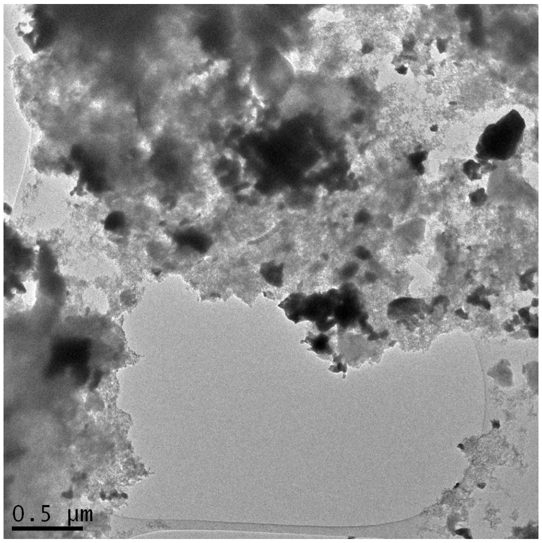

Figure S1 Low-magnification TEM image of **1**.

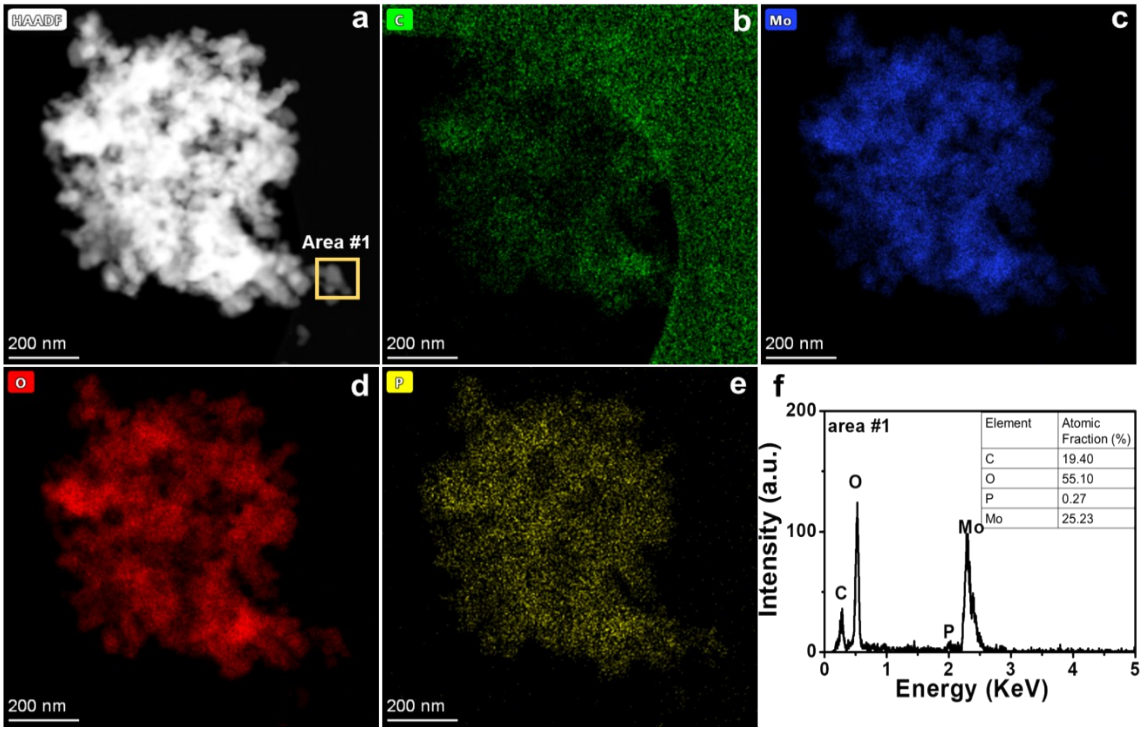

Figure S2 HAADF-STEM (a) and corresponding EDS mapping (b-e) of **1**, showing the homogeneous dispersion of Mo, P and O on carbon matrix. (f) Integrated EDS spectrum and quantitative element analysis collected from the area #1 shown in (a). The quantitative EDX analysis shows a stoichiometry of about 1:2 between Mo and O and therefore confirms the presence of MoO_2.

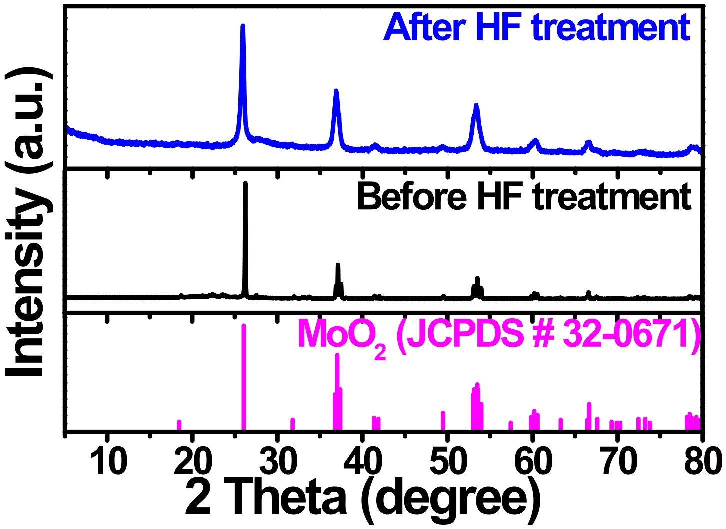

Figure S3 PXRD spectra of **1** before and after HF treatment, which shows similar structural features for MoO_2.

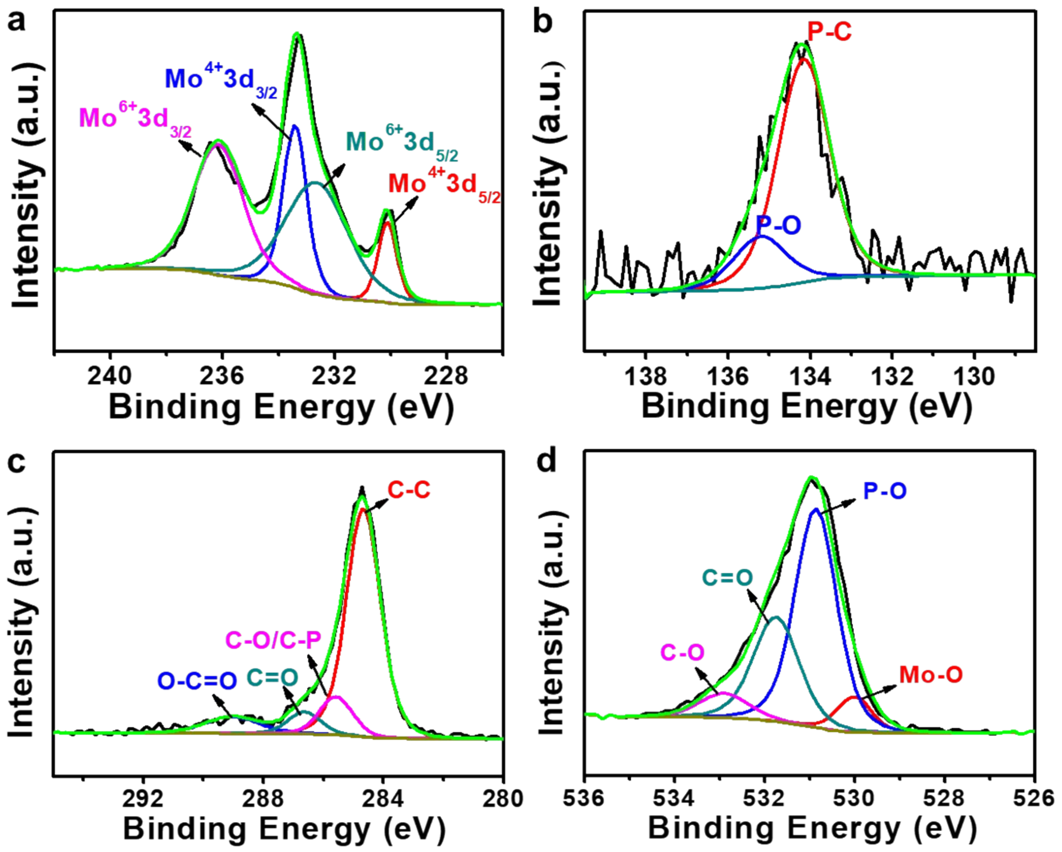

Figure S4 High-resolution XPS spectra for 1. a, Mo 3d. b, P 2p. c, C 1s and d, O 1s.

Figure S4 shows the high-resolution Mo 3d, P 2p, C 1s and O 1s XPS spectra for **1**. Deconvolution for Mo 3d (Figure S4a) gives four peaks assigned to Mo $3d_{5/2}$ (230.1 eV) and Mo $3d_{3/2}$ (233.4 eV) for Mo (IV) included in the MoO_2, and Mo $3d_{5/2}$ (232.6 eV) and Mo $3d_{3/2}$ (236.1 eV) for Mo (VI) assigned to MoO_3, formed by surface oxidation of the metastable MoO_2 in air.[6,7] For P 2p (Figure S4b), deconvolution gives two peaks assigned to P-C (134.2 eV) and P-O (135.2 eV),[8] suggesting that P was incorporated into the carbon framework. For C 1s (Figure S4c), four peaks centered at 284.7, 285.6, 286.6 and 289.1 eV can be attributed to C-C, C-O/C-P, C=O and O-C=O, respectively,[8] further confirming that P was integrated into the carbon framework. For O 1s (Figure S4d), the broad peak can be deconvoluted into four peaks centered at 530.0, 530.9, 531.7 and 532.9 eV assigned to Mo-O, P-O, C=O and C-O.[9]

Table S1 The binding energies (eV) of Mo 3d, P 2p, C 1s, O 1s and N 1s for different materials

		Composite **1**	Composite **2**	Composite **3**
Mo 3d	Mo^{IV} 3d $_{3/2}$	233.4	234.0	234.0
	Mo^{IV} 3d $_{5/2}$	230.1	230.8	230.8
	Mo^{VI} 3d $_{3/2}$	236.1	236.0	236.0
	Mo^{VI} 3d $_{5/2}$	232.6	232.9	232.8
P 2p	P-C	134.2	133.8	133.8
	P-O	135.2	135.0	135.2
C 1s	C-C	284.7	284.7	284.8
	C-O/C-P (/C=N)	285.6	285.7	285.5
	C=O (/C-N)	286.6	286.8	286.7
	O-C=O	289.1	288.7	288.1
O 1s	P-O	530.9	531.0	530.7
	C=O	531.7	531.7	531.7
	C-O	532.9	532.9	533.1
	Mo-O	530.0	530.4	530.1
N 1s	Pyridinic N	-	-	398.6
	Pyrrolic N	-	-	399.9
	Graphitic N	-	-	401.0

Table S2 Nitrogen sorption analyses for different materials

**Samples**	**BET specific surface area (m^2/g)**	**Pore volume (cm^3/g) by DFT calculation**	**Pore diameter (nm) by DFT calculation**
**1**	52.23	0.097	2.769
**2**	740.71	1.060	2.769
**3**	822.74	0.660	1.543
**MC**	853.53	1.521	5.199
**OMC**	690.93	1.293	5.199
**NMC**	431.69	0.856	4.543

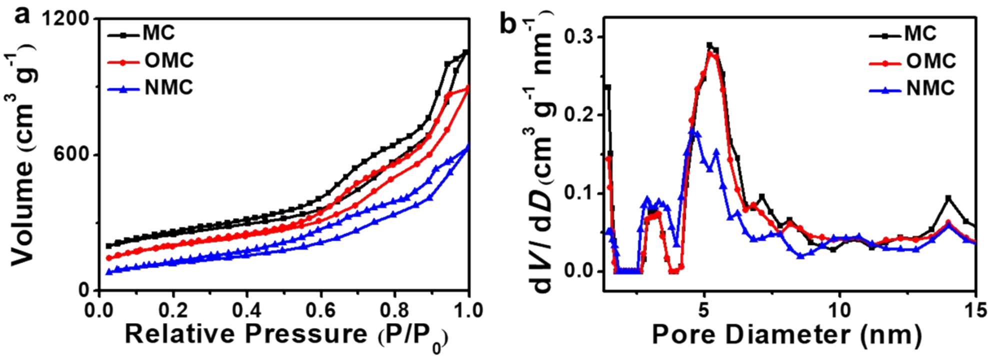

Figure S5 Nitrogen adsorption-desorption analysis. a, Nitrogen adsorption-desorption isotherms and b, the corresponding DFT pore size distributions of MC, OMC and NMC.

The N_2 sorption isotherm curves for all samples (**1**, **2** and **3**) displayed a similar IV-type isotherm characteristic for micro/mesoporous materials (Figure 2b of main manuscript). This is in agreement with the reference samples prepared by classical methods (**MC**, **OMC** and **NMC**, see Figure S5a). Compared with pure **MC**, **1** exhibited a significantly lower Brunauer–Emmett–Teller (BET) surface area. The BET surface area of **2** (740.7 m^2/g) and **3** (822.7 m^2/g) (*i.e.* after HNO_3 oxidation) are increased compared with **1** (52.2 m^2/g), indicating significant structural changes and increasing porosity upon the oxidative treatment. In contrast, for the reference samples prepared by classical procedures, the BET surface area (Figure S5a) of **OMC** (690.9 m^2/g) and **NMC** (431.7 m^2/g) decreased notably compared with **MC** (853.5 m^2/g), indicating that the presence of the Mo oxide might play a role in porosity generation. The pore size distributions of all samples were calculated by density functional theory (DFT) modelling, see Figure 2c of main manuscript and Figure S5b. While **MC** only showed mesopores, **1** showed the presence of micropores together with a low cumulative pore volume. In contrast, **2** and **3** feature mesoporosity also (Figure 2c of main manuscript) which leads to the higher overall porosity observed and a larger cumulative pore volumes compared with **1**.

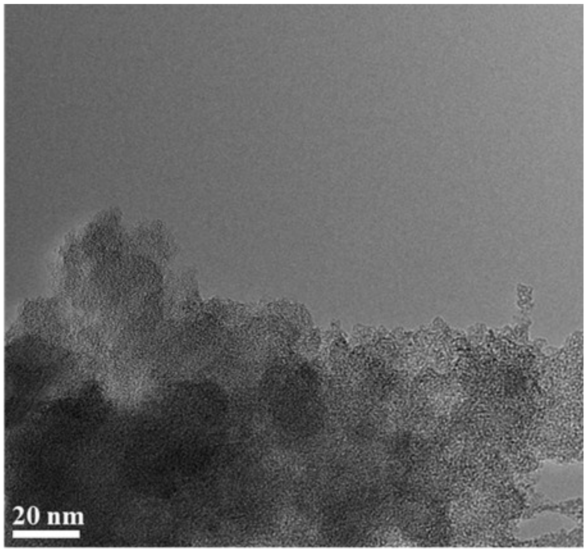

Figure S6 Representative overview TEM image of **2**.

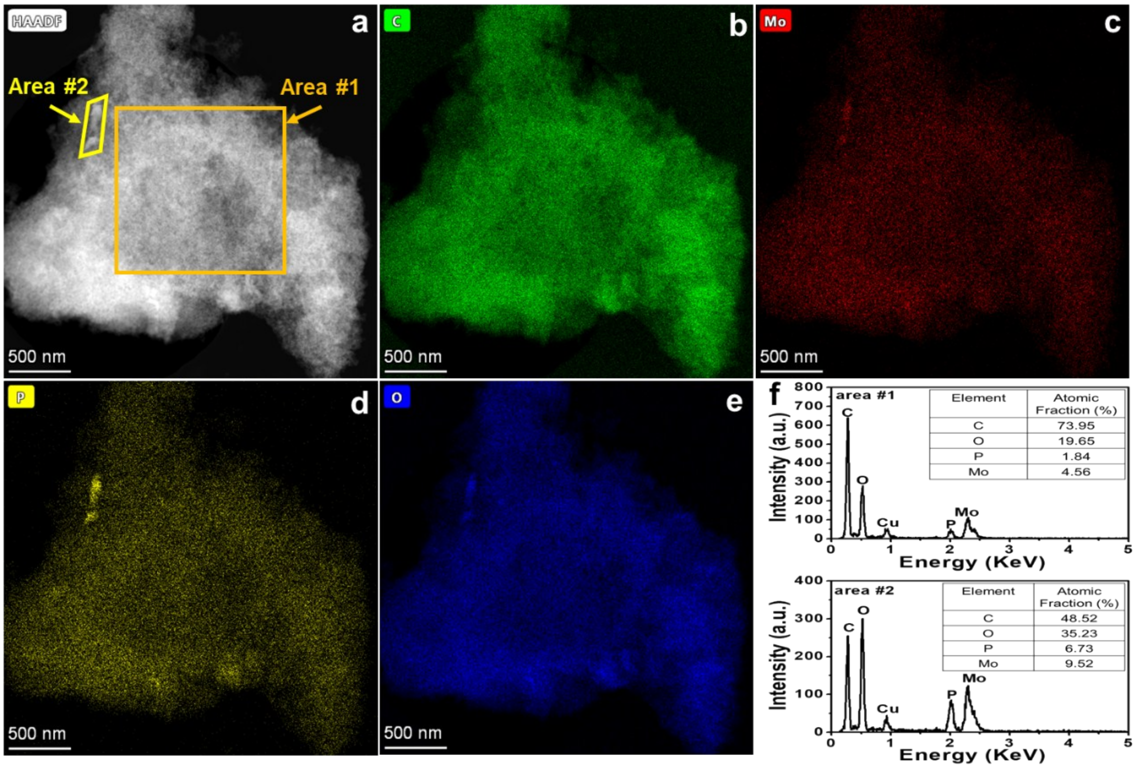

Figure S7 HAADF-STEM (a) and corresponding EDS mapping (b-e) of **2**, showing the homogeneous dispersion of Mo, P and O on the carbon matrix. (f) Integrated EDS spectra together with quantitative analysis collected from the area #1 (top) and area #2 (bottom) shown in (a). Area #2 in (a) shows amorphous aggregates that contain higher amounts of Mo, P and O.

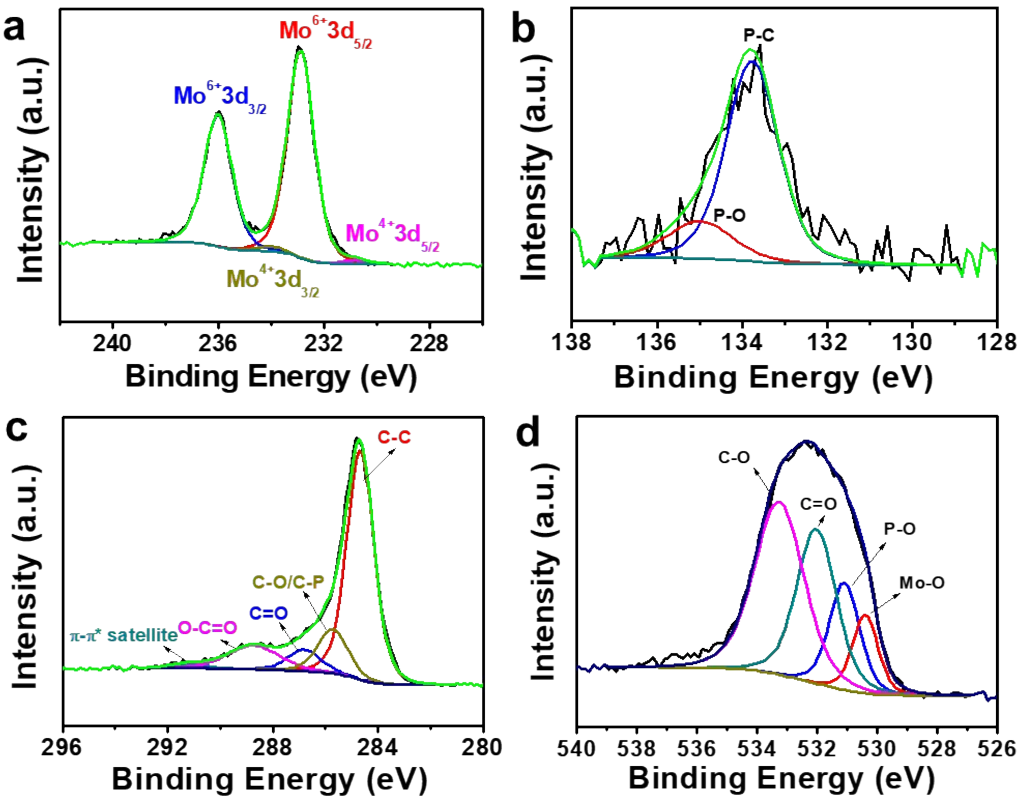

Figure S8 High resolution XPS spectra for **2**. a, Mo 3d. b, P 2p. c, C1s and d, O1s.

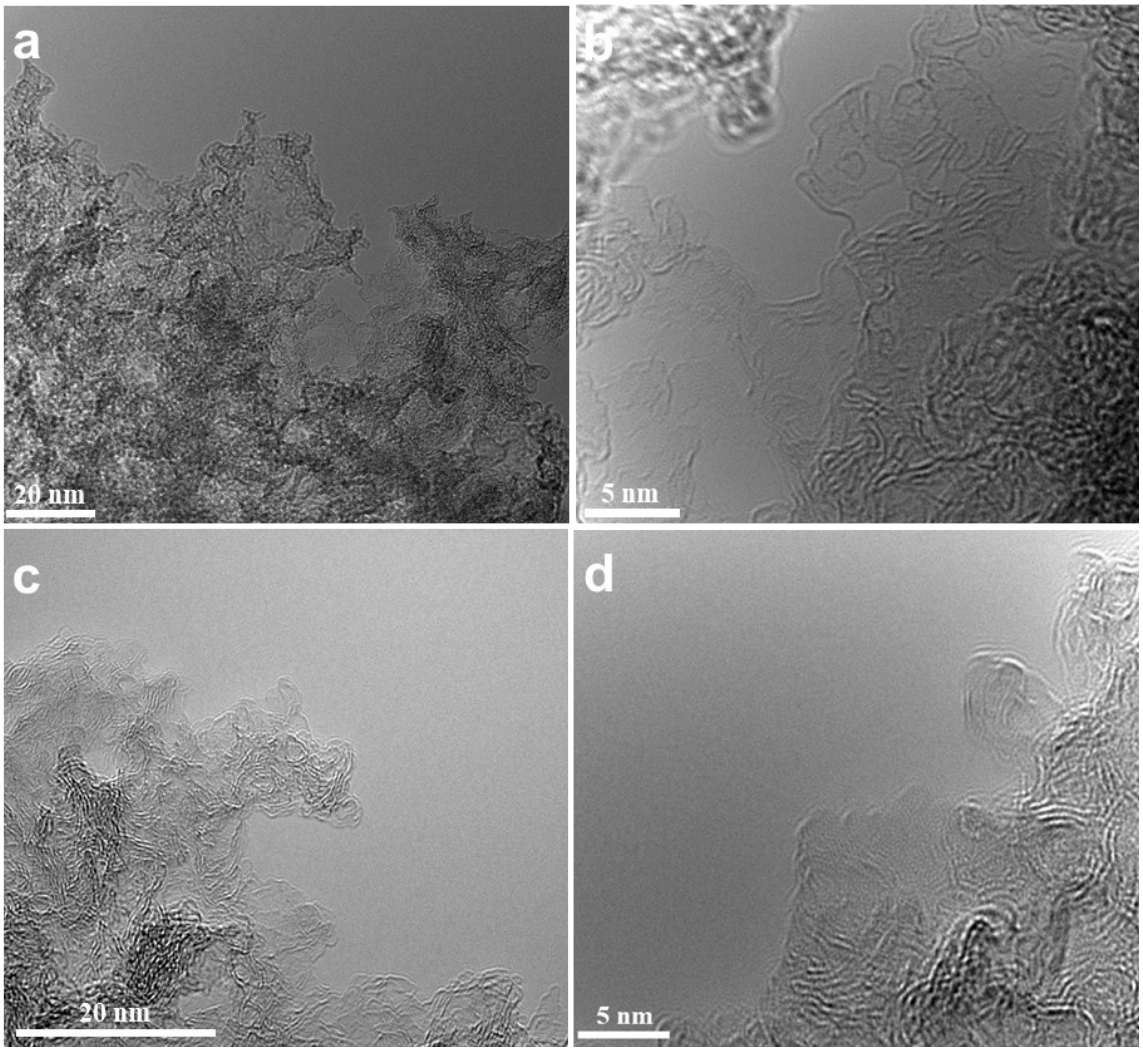

Figure S9 Representative TEM and HRTEM images of MC (a,b) and NMC (c,d).

Pure **MC** reference materials were formed by the classical synthetic route where sulfuric acid was used as carbonization catalyst. Figure S9 shows the TEM and HRTEM images of **MC** (Figure S9a,b) and NMC (Figure S9c,d). Note that no crystalline particles or metal-oxo clusters were observed. Instead, we only observed a porous carbon structure.

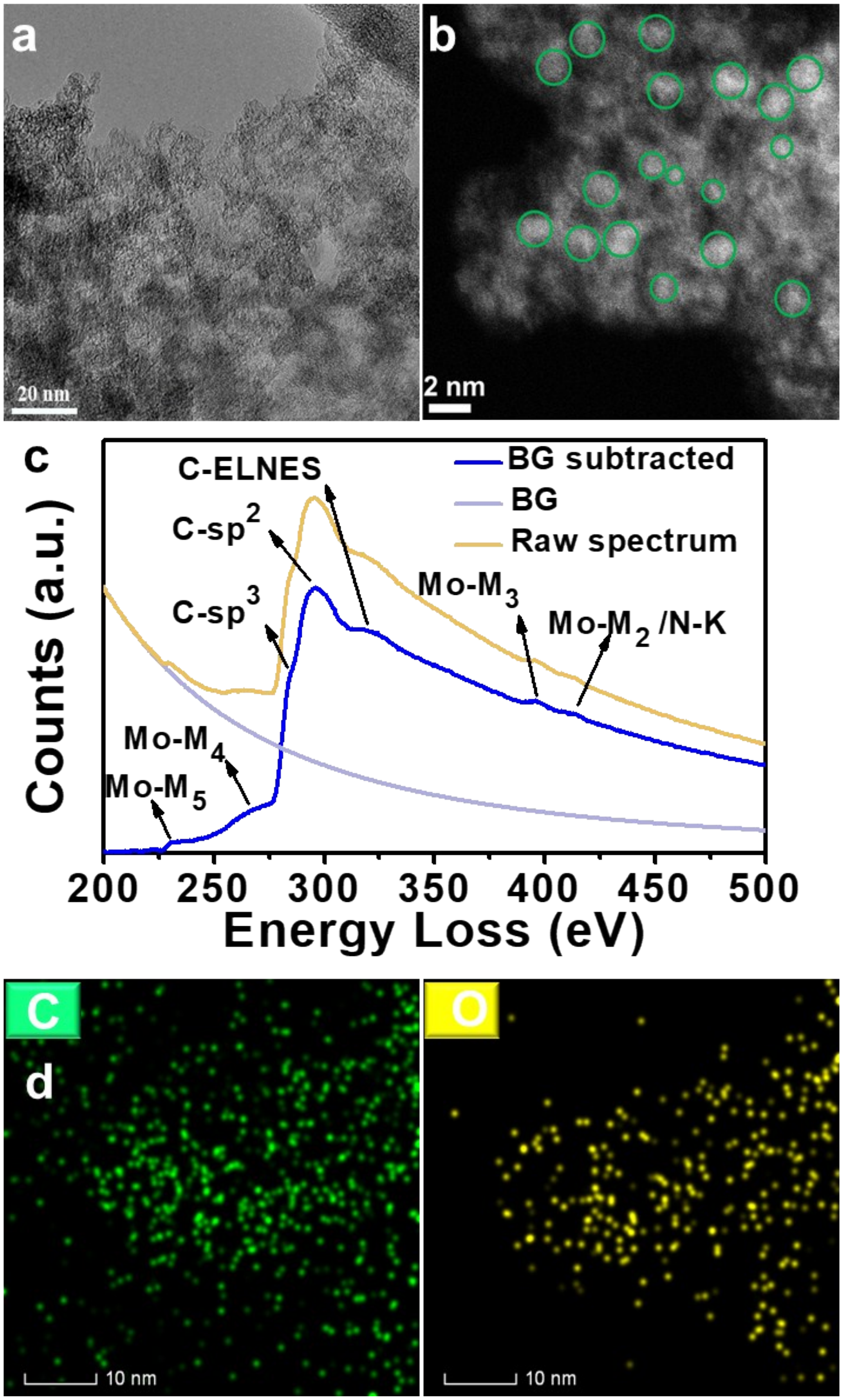

Figure S10 Morphology and chemical structure of **3**. a, TEM image of **3**. b, HAADF-STEM image of **3**. c, EELS spectra recorded from the HAADF-STEM image**.** BG = background; C-ELNES = carbon fine structure electron loss near edge structure. d, STEM-EDS elemental mapping of **3**, showing the homogeneous distribution of C and O (for other elements see main manuscript).

Table S3. Results of linear combination fitting to the Mo K-edge XANES.

Composite	MoO_2 Fraction (%)	MoO_3 Fraction (%)
**1**	74	26
**2**	12	88
**3**	14	86

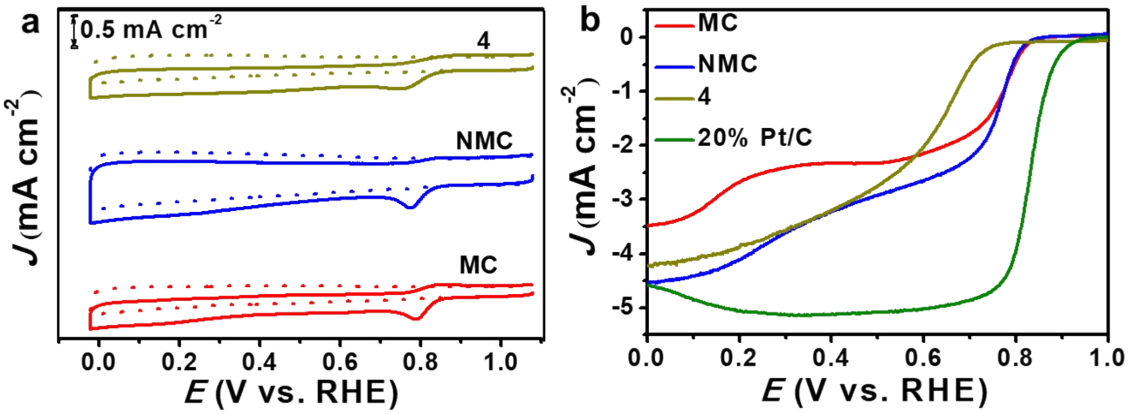

Figure S11 ORR activity studied by using CV and RDE techniques. a, CV curves of MC, NMC and 4 in Ar- (dotted line) or O_2-saturated (solid line) 0.1 M aqueous KOH at a sweep rate of 5 mV s^{-1}. b, LSV polarization curves of MC, NMC, **4** and 20% Pt/C in O_2-saturated 0.1 M aqueous KOH at a sweep rate of 5 mV s^{-1}, the rotating speed was 1600 rpm.

In comparison, CV and LSV results (Figure S11) show that the **NMC** has similar activity to pure **MC**, indicating that the oxidation of **MC** by HNO_3 and the introduction of nitrogen did not lead to reactivity increases.

Table S4 Electrocatalytic ORR activity of the different catalysts studied based on CV analysis

Samples	E_p (V vs. RHE)	J_p (mA cm^{-2})
**1**	0.62	-0.30
**2**	0.78	-0.31
**3**	0.82	-0.94
**4**	0.74	-0.36
**MC**	0.79	-0.40
**NMC**	0.77	-0.73

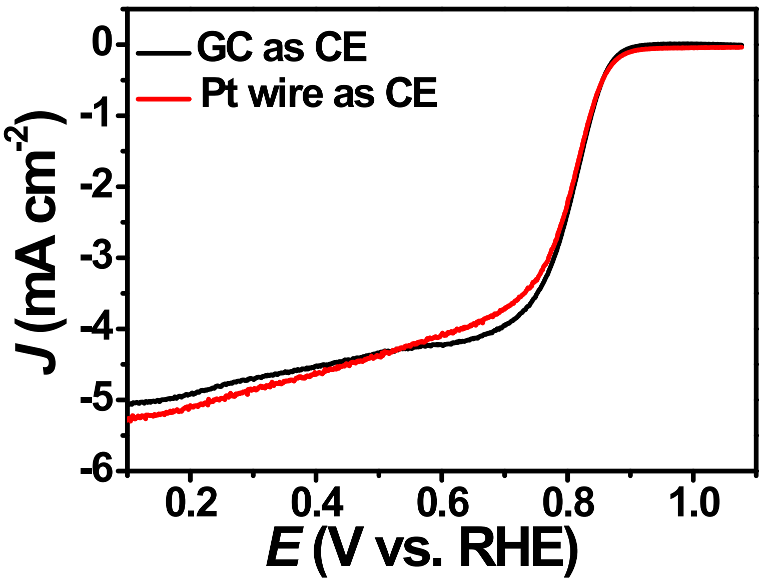

Figure S12 LSV curves of **3** in O_2-saturated 0.1 M aqueous KOH at a sweep rate of 5 mV s^{-1} and rotating speed of 1600 rpm, by using glassy carbon (GC) and Pt wire as counter electrodes. Freshly prepared catalyst-modified electrodes were prepared and used for the two measurements.

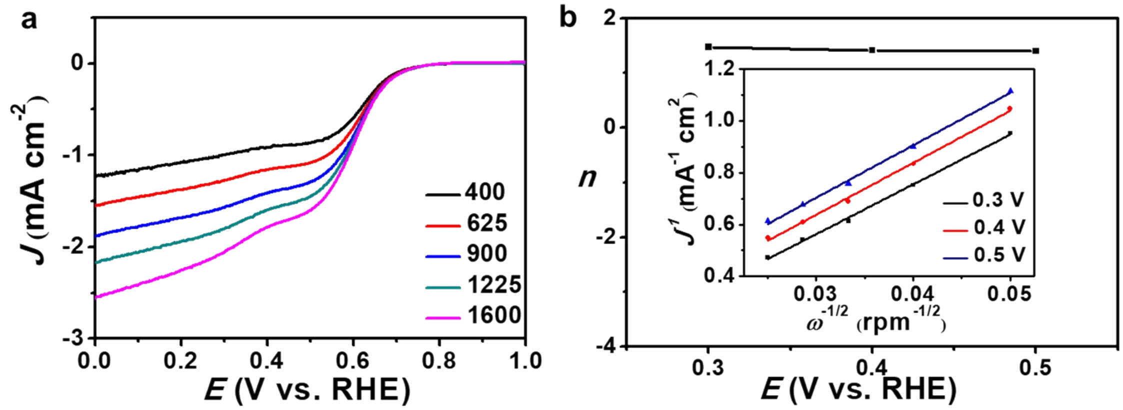

Figure S13 ORR activity studied by RDE analysis. a, LSV polarization curves of **1** in O_2-saturated 0.1 M aqueous KOH at a sweep rate of 5 mV s^{-1} at varying rotation rates (400-1600 rpm). b, electron transfer number (*n*) for **1** at different potentials obtained from K-L plots (see insert).

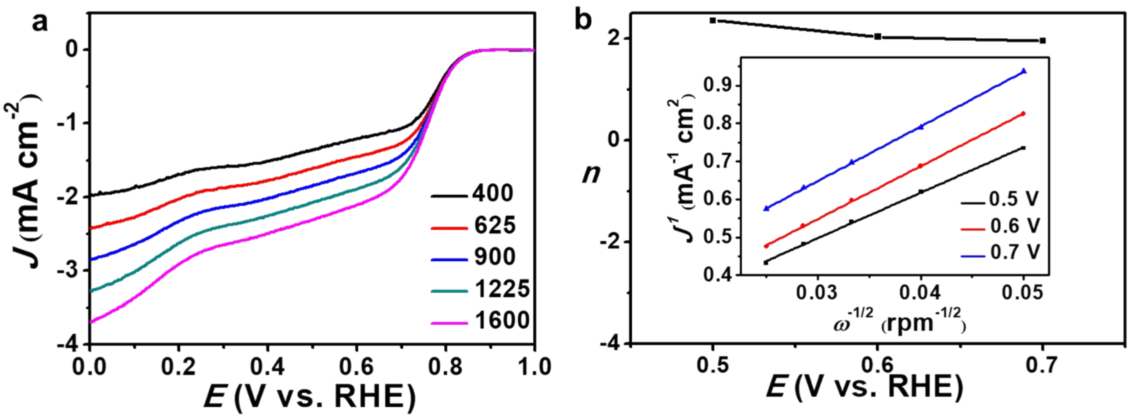

Figure S14 ORR activity studied by RDE analysis. a, LSV polarization curves of a **2** in O_2-saturated 0.1 M aqueous KOH at a sweep rate of 5 mV s^{-1} with varying rotation rates (400-1600 rpm). b, electron transfer number (*n*) for a **2** at different potentials obtained from K-L plots (see insert).

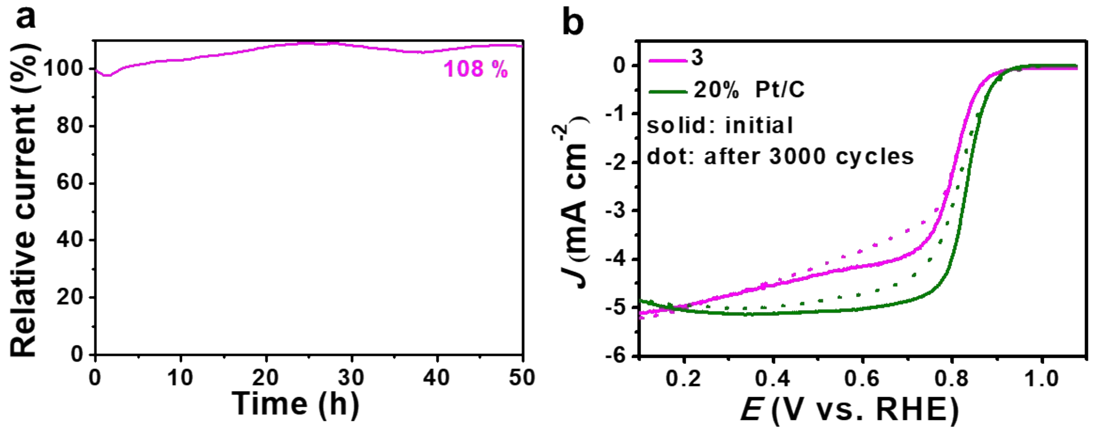

Figure S15 a, Chronoamperometry of **3** modified RDE at 0.7 V *vs.* RHE in 0.1 M aqueous KOH with continuous O_2 bubbling at 200 rpm for 50 h. b, LSV polarization curves of **3** and 20% Pt/C before and after 3000 cycles between 0.6 and 1.0 V (scan rate 200 mV s^{-1} in O_2-saturated 0.1 M aqueous KOH).

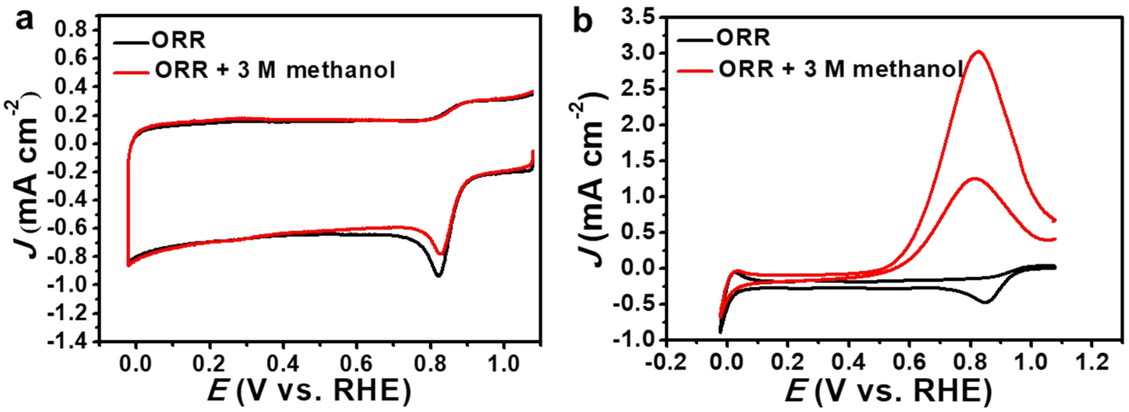

Figure S16 Methanol tolerance tests. CV curves of a, **3** and b, 20% Pt/C in O_2-saturated 0.1 M aqueous KOH (black) and O_2-saturated 0.1 M aqueous KOH with 3 M methanol (red) solution at a scan rate of 5 mV s^{-1}.

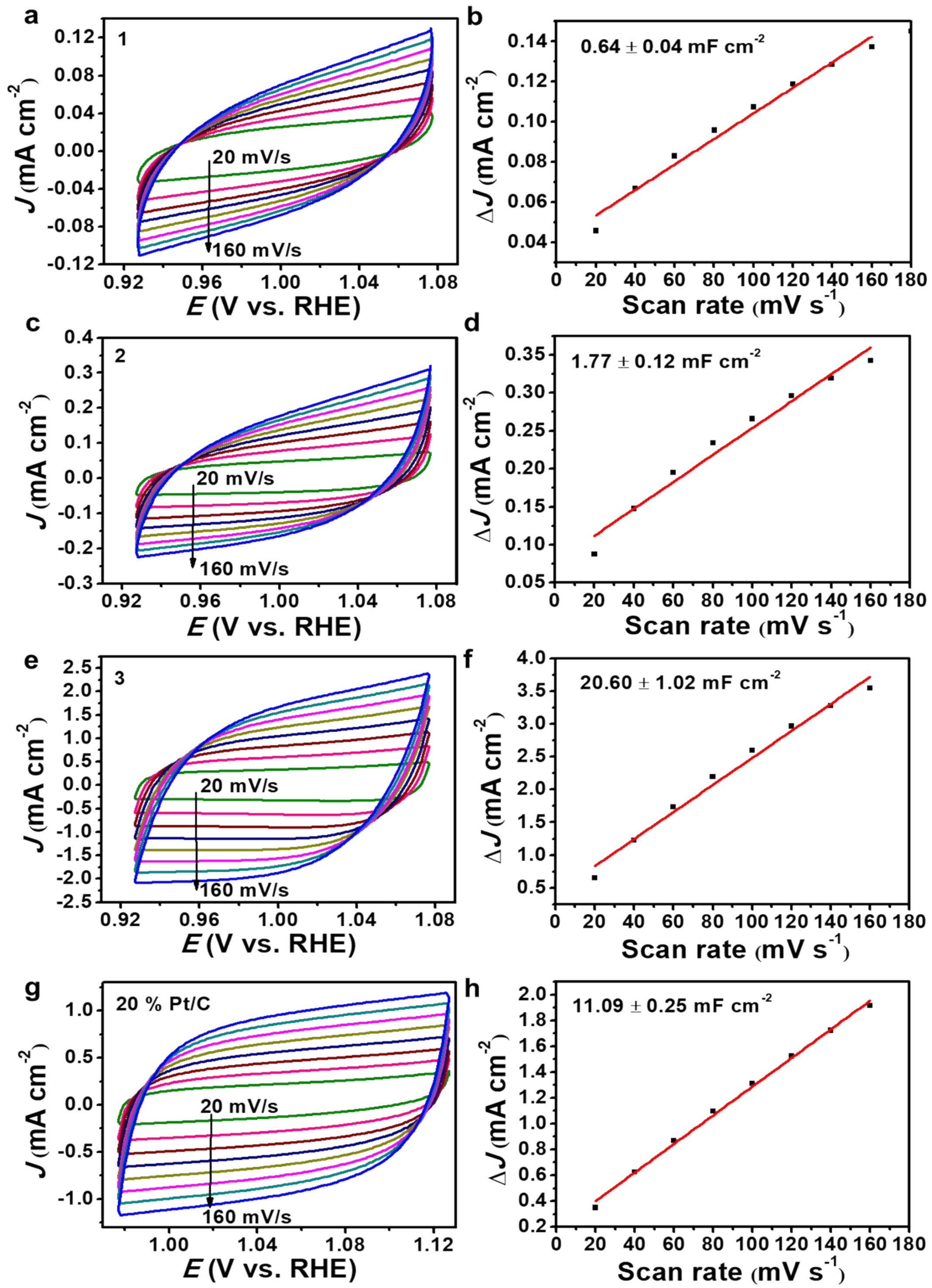

Figure S17 ECSA study of the catalysts. a,c,e,g CV curves for **1**, **2**, **3** and 20% Pt/C at various scan rates. b,d,f,h The corresponding linear fitting plots of differences in current density ($\Delta J = J_a - J_c$) at a potential of 1.0 V for **1**, **2** and **3**, and 1.05 V for 20% Pt/C depending on the scan rate. J_a: anodic current density; J_c: cathodic current density.

ECSA is calculated based on the equations shown below:[10]

(1) $\Delta J = \gamma \times C_{dl}$

(2) ECSA = C_{dl}/C_s

(3) C_s = 40 μF/cm^2 per cm^2

where γ is scanning rate, ΔJ (= J_a - J_c) is charging current density differences, C_{dl} is double-layer capacitance and C_s is specific capacitance of the catalyst.[11]

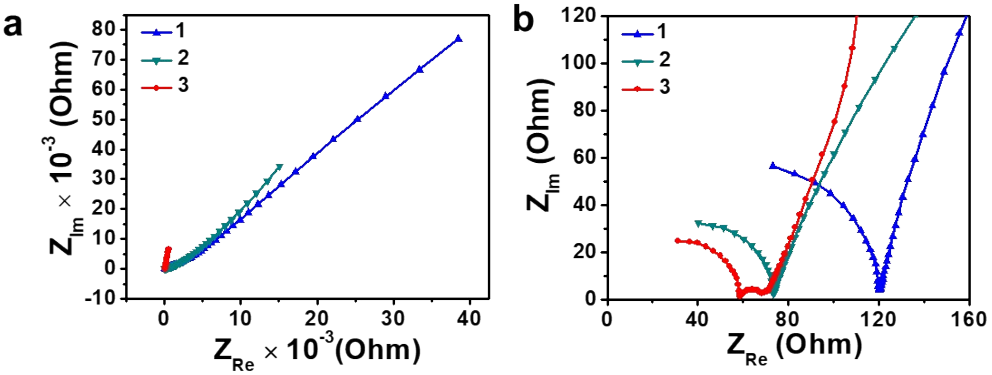

Figure S18 EIS analyses of the composite electrocatalysts. a, Nyquist plots of **1**, **2** and **3** obtained by applying a sine wave with potential amplitude of 5.0 mV over the frequency range 0.01 Hz–1000 kHz. b, Corresponding high-frequency area of the Nyquist plots.

Figure S18a shows the Nyquist plots of **1**, **2** and **3** obtained by applying a sine wave with amplitude of 5.0 mV over the frequency range 0.01 Hz–1000 kHz. The equivalent series resistances extracted at 10 Hz of **1**, **2** and **3** are 1070, 610 and 76 Ohm, respectively. The Nyquist plots in the high-frequency region (Figure S18b) show that the diameter of the semicircle for **3** is smaller than that of **1** and **2**. The calculated charge-transfer resistances (R_{ct}) are shown in Table 1 of the main manuscript. These data indicate that **3** has the lowest charge-transfer resistances which is beneficial for the catalysis.

4. Supplementary references

1 J. J. Han, N. Li and T. Y. Zhang, *J. Power Sources*, 2009, **193**, 885–889.

2 A. J. Bard and L. R. Faulkner, *Electrochemical methods: fundamentals and applications*, John Wiley & Sons, New York, 2000.

3 S. Wang, D. Yu and L. Dai, *J. Am. Chem. Soc.*, 2011, **133**, 5182–5185.

4 L. Hao, S. Zhang, R. Liu, J. Ning, G. Zhang and L. Zhi, *Adv. Mater.*, 2015, **27**, 3190–3195.

5 K. Persson, 2014.

6 Y. Jin, H. Wang, J. Li, X. Yue, Y. Han, P. K. Shen and Y. Cui, *Adv. Mater.*, 2016, **28**, 3785–3790.

7 Y. Sun, X. Hu, W. Luo and Y. Huang, *ACS Nano*, 2011, **5**, 7100–7107.

8 Y. J. Tang, M. R. Gao, C. H. Liu, S. L. Li, H. L. Jiang, Y. Q. Lan, M. Han and S. H. Yu, *Angew. Chem. Int. Ed.*, 2015, **54**, 12928–12932.

9 A. Bhaskar, M. Deepa and T. Narasinga Rao, *ACS Appl. Mater. Interfaces*, 2013, **5**,

2555–2566.
10 C. C. L. McCrory, S. Jung, J. C. Peters and T. F. Jaramillo, *J. Am. Chem. Soc.*, 2013, **135**, 16977–16987.
11 J. Kibsgaard and T. F. Jaramillo, *Angew. Chem. Int. Ed.*, 2014, **53**, 14433–14437.
